# A Combined Score of Circulating miRNAs Allows Outcome Prediction in Critically Ill Patients

**DOI:** 10.3390/jcm8101644

**Published:** 2019-10-09

**Authors:** Christoph Roderburg, Fabian Benz, Alexander Koch, Sven H. Loosen, Martina Spehlmann, Mark Luedde, Alexander Wree, Mihael Vucur, Christian Trautwein, Frank Tacke, Tom Luedde

**Affiliations:** 1 Department of Medicine III, University Hospital RWTH Aachen, Pauwelsstrasse 30, 52074 Aachen, Germany; fabian.benz@charite.de (F.B.); aKoch@ukaachen.de (A.K.); sloosen@ukaachen.de (S.H.L.); alexander.wree@charite.de (A.W.); mvucur@ukaachen.de (M.V.); ctautwein@ukaachen.de (C.T.); frank.tacke@charite.de (F.T.); tluedde@ukaachen.de (T.L.); 2 Department of Gastroenterology/Hepatology, Campus Virchow Klinikum and Charité Campus Mitte, Charité University Medicine Berlin, Augustenburger Platz 1, 13353 Berlin, Germany; 3 Department of Cardiology and Angiology, University of Kiel, Schittenhelmstrasse 12, 24105 Kiel, Germany; martina.spehlmann@uksh.de (M.S.); Mark.Luedde@uksh.de (M.L.)

**Keywords:** miRNA, biomarker, critical illness, sepsis, prognosis

## Abstract

Background and aims: Identification of patients with increased risk of mortality represents an important prerequisite for an adapted adequate and individualized treatment of critically ill patients. Circulating micro-RNA (miRNA) levels have been suggested as potential biomarkers at the intensive care unit (ICU), but none of the investigated miRNAs displayed a sufficient sensitivity or specificity to be routinely employed as a single marker in clinical practice. Methods and results: We recently described alterations in serum levels of 7 miRNAs (miR-122, miR-133a, miR-143, miR-150, miR-155, miR-192, and miR-223) in critically ill patients at a medical ICU. In this study, we re-analyzed these previously published data and performed a combined analysis of these markers to unravel their potential as a prognostic scoring system in the context of critical illness. Based on the Youden’s index method, cut-off values were systematically defined for dysregulated miRNAs, and a “miRNA survival score” was calculated. Patients with high scores displayed a dramatically impaired prognosis compared to patients with low values. Additionally, the predictive power of our score could be further increased when the patient’s age was additionally incorporated into this score. Conclusions: We describe the first miRNA-based biomarker score for prediction of medical patients’ outcome during and after ICU treatment. Adding the patients’ age into this score was associated with a further increase in its predictive power. Further studies are needed to validate the clinical utility of this score in risk-stratifying critically ill patients.

## 1. Introduction

In the last years, many circulating biomarkers have been evaluated for their potential to categorize critically ill patients according to their estimated prognosis during or after intensive care unit (ICU) treatment. In the context of “personalized medicine” approaches, such information were suggested to define individualized ICU management strategies based on early risk stratification, as was recently proposed in the Surviving Sepsis Campaign [1]. However, it became apparent that both the specificity and sensitivity of most (if not all) single markers in the context of critical illness are too low to justify their use in clinical routine [2]. In the absence of a single accepted biomarker, many authors have proposed not only attempting to identify new classes of biomarkers, but also to combine several clinical or laboratory biomarkers to improve their potential regarding the prediction of ICU patients’ prognosis [3,4,5].

miRNAs are small non-coding RNA molecules (~22 nucleotides) that repress the translation of their target mRNAs by binding to their 3′-untranslated region (UTR), and thereby play an important role in various physiological and pathological processes [6]. Besides their role in the regulation of gene expression, circulating miRNAs have emerged as a new class of biomarkers for various diseases such as organ fibrosis [7,8], systemic inflammation, and sepsis [9]. However, despite an initial enthusiasm in the field, miRNA-based biomarkers have not yet entered clinical routine due to incoherent normalization and non-standardized sample handling protocols. Moreover, it was recognized that similar to protein-based biomarkers, concentrations of circulating miRNAs also only display a limited diagnostic and prognostic power when used as stand-alone biomarkers in the setting of critical illness [10].

In the present study, we demonstrate that a composite score containing selected miRNAs has a considerably higher performance in identifying patients with a favorable prognosis during or after ICU treatment compared to individual miRNAs. Interestingly, the integration of the patient’s age into this miRNA-based score leads to a further increase in its sensitivity and specificity.

## 2. Patients and Methods

### 2.1. Study Design

The cohorts of patients for analysis of single miRNAs are described elsewhere in detail [9,11,12,13,14,15]. The cohort of patients included in the present analysis is described within Table 1 and Table 2. All patients included in the study provided written informed consent, and the ethics committee approved this consent procedure. The study protocol is in line with Declaration of Helsinki and was approved by the local ethics committee (ethics committee of the University Hospital Aachen, RWTH-University, Aachen, Germany, reference number EK 150/06). All patients were treated in accordance with current guidelines for treatment of sepsis (Surviving Sepsis Campaign) and specific guidelines of the respective boards. The clinical course of patients was followed up for a period of three years by directly contacting the patients, the patients’ relatives, or their primary care physician.

### 2.2. Extraction of miRNA and qPCR

MiRNA extraction and qPCR was described elsewhere in detail [9,11,12,13,14,15]. In brief, blood was collected using serum monovettes (Sarstedt, Nümbrecht, Germany), centrifuged for 8 min at 2000× *g* using a Rotixa 50 centrifuge (Hettich, Tuttlingen, Germany). Then, 400 µL serum was spiked with miScript miRNA mimic SV40 (Qiagen 2 µM, 1 µL/100 µL serum) for sample normalization. Afterwards, 800 µL phenol (Qiagen, Hilden, Germany) and 200 µL chloroform were added to the sample and mixed vigorously for 15 s, followed by an incubation at room temperature for 10 min. Samples were centrifuged for 15 min at 12,000× *g* until complete phase separation. The aqueous phase was precipitated with 500 µL 100% isopropanol and 2 µL glycogen (Fermentas, St. Leonroth, Germany) overnight at −20 °C. After centrifugation at 4 °C for 30 min (12,000× *g*), the pellets were washed once with 70% ethanol. Precipitated RNA was resuspended in 30 µL RNase-free water (Ambion, Austin, TX, USA). For quantitative real-time polymerase chain reaction (PCR), 5 µL of extracted total RNA was used to synthesize complementary deoxyribonucleic acid (cDNA) utilizing a miScript Reverse Transcriptase Kit (Qiagen, Hilden, Germany) according to the manufacturer’s protocol. cDNA samples (2 µL) were used for quantitative real-time PCR in a total volume of 25 µL using the miScript SYBR Green PCR Kit (Qiagen) and miRNA specific primers (Qiagen, primer sequences available online) on a qPCR machine (Applied Biosystems 7300 Sequence Detection System, Applied Biosystems, Foster City, CA, USA). Data were generated and analyzed using the SDS 2.3 and RQ manager 1.2 software packages.

### 2.3. Statistical Analysis

All statistical analyses were performed with SPSS (SPSS, Chicago, IL, USA) as described previously [16]. Data are displayed as median and range considering the skewed distribution of most parameters. Gaussian distribution was tested with Kolmogorov–Smirnov test. Differences between two groups were assessed by Student’s *t*-test or Wilcoxon rank-sum test, and multiple comparisons between more than two groups were conducted by ANOVA with Bonferroni test or Dunns test for post-hoc analysis. Box and whisker plot graphics illustrate a statistical summary. Here, the box represents the median with interquartile range (IQR), and the “whiskers” include all values smaller than the upper quartile plus 1.5*IQR and larger than the lower quartile minus 1.5*IQR. Values outside of the whiskers are displayed as separate points and represent outliers. All values, including outliers, were included for statistical analyses. Correlations between variables were analyzed using the Spearman correlation test. Receiver operating characteristic (ROC) curve analysis and the derived area under the curve (AUC) statistic provide a global and standardized appreciation of the accuracy of a marker or a composite score for predicting an event. ROC curves were generated by plotting sensitivity against 1-specificity. Optimal cut-off values were calculated by maximizing the Youden’s index, which is defined as sensitivity + specificity − 1 [17]. Kaplan–Meier curves were plotted to display the impact on survival and between-group differences were assessed using the log-rank test. Cox regressions were used to identify factors predicting ICU mortality or overall mortality. All reported *p*-values are two-tailed, and a *p*-value of less than 0.05 is considered statistically significant.

## 3. Results

### 3.1. Alterations of Circulating miRNA Concentrations in Critically Ill Patients

We recently described alterations in serum levels of miR-122, miR-133a, miR-143, miR-150, miR-155, miR-192, and miR-223 in patients that were treated on our medical intensive care unit [9,11,12,13,14,15]. In this study, we re-analyzed these previously published data and performed a combined analysis of these markers in critically ill patients. Only patients with valid results for all seven miRNAs were included in the present analysis, which was the case for 204 patients (Table 1 and Table 2). We found significantly higher levels of miR-122, miR-133a, miR-155, and miR-192 in critically ill patients when compared to healthy controls, while levels of miR-150 and miR-223 were slightly lower in the patient group, which is in line with our previous reports (Table 3). Of the 204 patients included in the present analysis, 127 fulfilled the Sepsis-3 criteria [18] at the time point of admission to the ICU (Table 2). In line with our previous results, only miR-133a levels were altered in patients with sepsis compared to patients with non-septic disease (Table 3). Out of the 204 patients, 45 died during ICU treatment and an additional 40 patients died during the long-term observation period (post-ICU/post-hospital). Similar to our previous reports, concentrations of miR-133a were significantly elevated, whereas levels of miR-143 as well as miR-223 were significantly decreased in patients who did not survive the ICU stay, and miR-133a (up) and miR-150 (down) displayed a significant deregulation in patients that displayed an unfavorable overall prognosis (Table 3).

### 3.2. A Three miRNA Score Predicts Patients’ Survival in ICU Treatment

**Figure 1:**
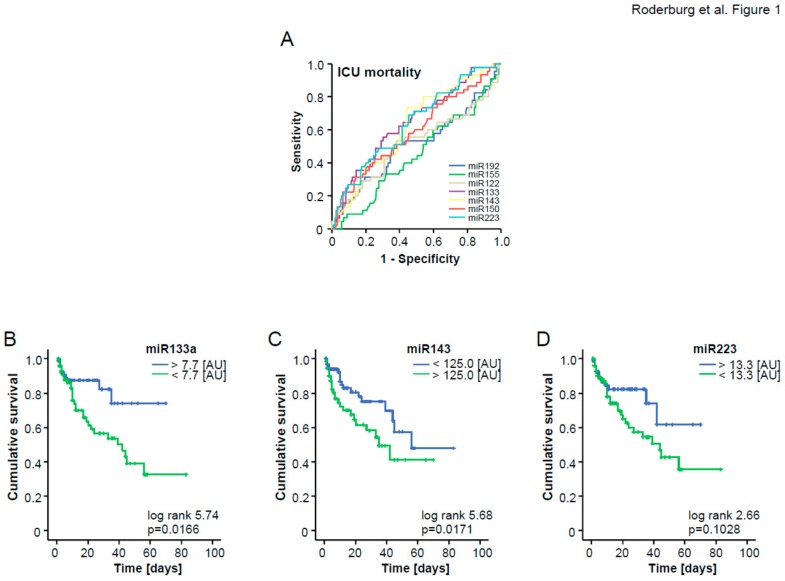
(**A**) ROC analysis comparing all seven single miRNAs at admission for ICU mortality. (**B**–**D**) Optimal cutoffs were calculated for miR-133a, miR-143, and miR-223 using Youden’s index to discriminate between ICU non-survivors and survivors. Kaplan–Meier curves with miR-133a, miR-143, and miR-223 as single biomarker are displayed.

**Figure 2:**
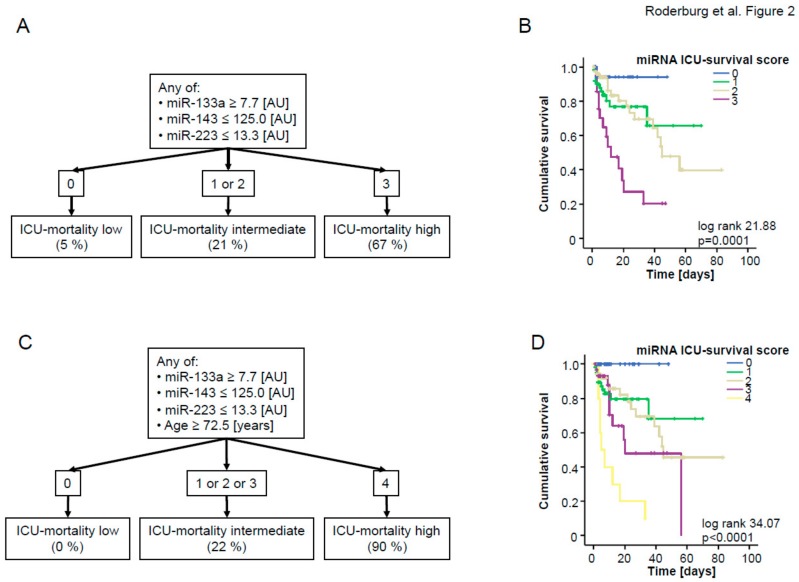
(**A**) The individual “miRNA survival score” was calculated as described in the text. In brief, patients with a higher (in the case of miR-133a) or lower (in the case of miR-143 and miR-223) levels than the respective cut-off were given one point for each individual dysregulated miRNA. All points were added to an “ICU survival score”, (**B**) Kaplan–Meier curve analysis of ICU patients demonstrates that patients with a high “ICU survival score” had an increased short-term mortality compared to other patients; *p*-values are given in the figure. (**C**) Age was included in the “ICU survival score”. Patients older than 72.5 years received one additional point. (**D**) Kaplan–Meier curve analysis of ICU patients demonstrates that patients with higher “ICU survival score + age” had an increased short-term mortality compared to other patients; *p*-values are given in the figure.

To analyze the potential of combining different miRNAs for the prediction of patients‘ prognosis, we next included those miRNAs that displayed a significant down-regulation in patients who did not survive ICU treatment (Table 3) into ROC curve analysis and calculated an “optimal” statistical cut-off value for each miRNA regarding the discrimination between survivors and non-survivors by using the Youden’s index method (Figure 1A). We next validated the respective cut-off values for each individual miRNA in Kaplan–Meier survival analysis, revealing clear differences in patients’ ICU survival when they were categorized using the calculated cut-offs (Figure 1B–D). Considering these results, we subsequently hypothesized that a combined score consisting of the significantly regulated miRNAs (miR-133a, miR-143, and miR-223) might outperform individual miRNA measurements regarding the discrimination between patients with a favorable prognosis during ICU treatment and those with a higher risk for ICU mortality. For this, we used the cut-off values derived from the area under the curve (AUC) analysis. Patients with a higher (in case of up-regulated miRNAs) or lower (in case of down-regulated miRNAs) concentration than the respective cut-off values were given one point for each individual dysregulated miRNA. Patients not fulfilling these criteria did not receive a point. All points were added to an “miRNA ICU survival score” (Figure 2A). By applying this score within our cohort, three different subgroups became apparent: Patients with 3 points in our survival score (“all miRNAs positive”) displayed an ICU mortality of 67%, while, in sharp contrast, patients with 0 points (“all miRNAs negative”) displayed a mortality rate of only 5%. Patients with 1 or 2 points had an intermediate risk of dying during their ICU stay (21%). In addition, we performed Kaplan–Meier curve analysis with our ICU score as the stratifying variable. This analysis confirmed that higher values were associated with an unfavorable prognosis (Figure 2B).

It was previously described that elderly patients have a disproportionately higher morbidity and mortality from sepsis [20]. We therefore hypothesized that adding the patient’s age to our score might further increase its accuracy to predict the individual ICU prognosis. We first calculated a cut-off value that best indicated the patient’s prognosis, as previously described (72.5 years). By including the patient’s age as an additional item (if age >72.5 years then 1 point, else 0 points), the performance of our score to estimate the patient’s outcome could be further increased: Patients with 4 points (“all miRNAs positive” and age >72.5 years) displayed an ICU mortality of 90%; patients with 0 points (“all miRNAs negative” and age <72.5 years) displayed a mortality of 0%, clearly highlighting that the use of our score allows to discriminate between low and high risk patients (Figure 2C,D).

Besides age, the presence of septic disease might also have a critical impact on the patient’s survival. To exclude that our ICU survival score was biased by sepsis, we analyzed the proportions of patients with lower or higher score values in the subgroup of patients with septic diseases and the subgroup of patients with a different etiology of critical illness. Importantly, we found no difference in the distribution of patients with higher or lower risk according to our score between these subgroups, highlighting that our score represents a reliable predictor of prognosis in medical ICU patients with or without sepsis (Supplementary Figure S1).

### 3.3. A Two miRNA Score Predicts Patients’ Long-Term Prognosis

**Figure 3:**
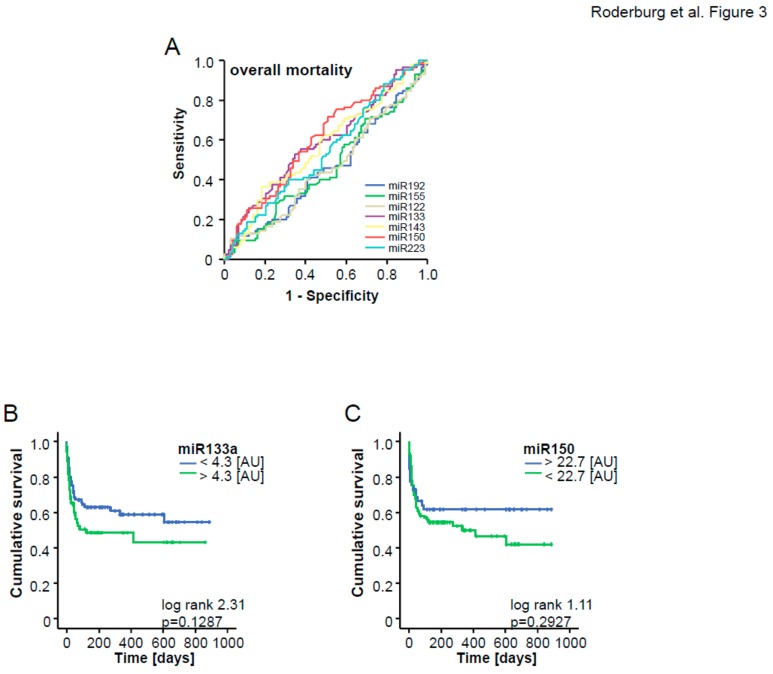
(**A**) ROC analysis comparing all miRNAs at admission for overall mortality. (**B**,**C**) optimal cut-offs were calculated for miR-133a and miR-150 using Youden’s index to discriminate between overall non-survivor and survivor. Kaplan–Meier curves of ICU patients with miR-133 and miR-150 as single biomarker.

**Figure 4:**
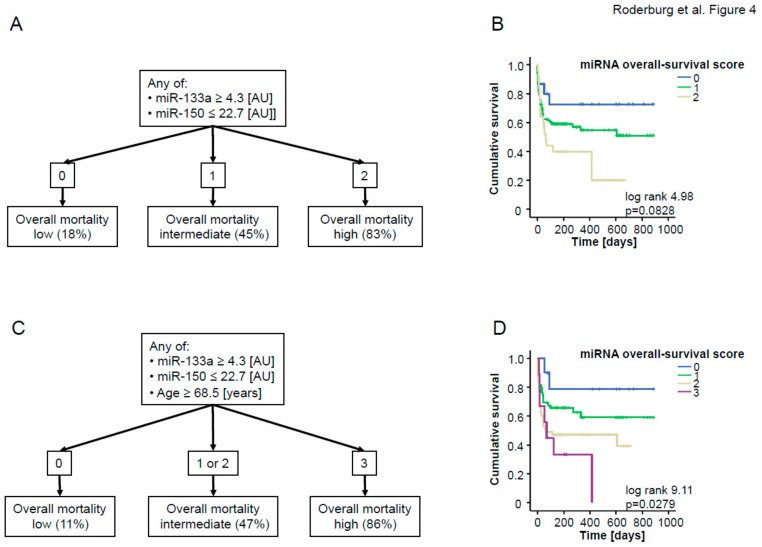
(**A**) The individual “overall survival score” was calculated as described in the text. In brief, patients with higher (in case of miR-133a) or lower (in case of miR-150) levels than the respective cut-off were given one point for each individual dysregulated miRNA. All points were added to an “overall survival score”, (**B**) Kaplan–Meier curve analysis of ICU patients demonstrates that patients with higher “overall survival scores” had an increased overall mortality compared to other patients; *p*-values are given in the figure. (**C**) Age was included into the score. Patients older than 68.5 years received one additional point. (**D**) Kaplan–Meier curve analysis of ICU patients demonstrates that patients with higher scores had an increased overall mortality compared to other patients; *p*-values are given in the figure.

Similar to the ICU survival score, we included those miRNA that displayed a significant down-regulation in patients who did not survive the long-term observation period compared to survivors into ROC curve analysis (Figure 3A) and calculated an “optimal” statistical cut-off value for each miRNA for distinguishing between survivors and non-survivors by using the Youden’s index method. We next validated these cut-off values for each individual miRNA in Kaplan–Meier survival analyses (Figure 3B,C) and generated an “miRNA overall survival score” by analyzing patients’ survival with respect to their individual score. If the miR-133a level was higher than 4.3 [AU], the patients were assigned 1 point, else 0 points, and similarly, if the miR-150 level was lower than 22.7 [AU], patients were assigned 1 point, else 0 points. Patients with 2 points in our “overall survival score” (“both miRNAs positive”) displayed a considerably higher overall mortality (83%) compared to patients with 0 points (“both miRNAs negative”, overall mortality: 18%), which was additionally confirmed by Kaplan–Meier curve analysis with our overall survival score as the stratifying variable (Figure 4A,B). This analysis confirmed that higher score values were associated with an unfavorable prognosis.

Again, by including the patient’s age as an additional item (if age >68.5 years then 1 point, else 0 points), the performance of our score to estimate patients’ overall survival could be further increased: Patients with 3 points (“all miRNAs positive” and age >68.5 years) displayed an overall mortality of 86%; patients with 0 points (“both miRNAs negative” and age <68.5 years) displayed a mortality rate of only 11%. In line, Kaplan–Meier curve analysis revealed a significantly impaired long-term outcome for patients who had a higher overall survival score (Figure 4C,D).

## 4. Discussion

In the present study, we demonstrate that a panel of miRNAs is predictive for the patients´ outcome during and after ICU treatment. Our multimarker approach improves the prognostic power in comparison to stand-alone marker approaches previously published in the field of miRNA research in critical care medicine. Together, such prognostic scoring systems could be highly relevant for guiding the individual treatment of critically ill patients at admission to the ICU.

Despite continuous advances in diagnostic modalities, the initial triage, as well as the diagnostic and therapeutic management of critically ill patients, have remained a major clinical challenge. In this context, besides specific triage systems, various laboratory markers potentially allowing decisions on the patients’ treatment and their individual clinical course were proposed. As such, apart from routinely used laboratory markers (e.g., C-reactive protein or procalcitonin; [2]), a variety of different experimental protein-based markers such as Ghrelin, Leptin, Resistin, and Osteopontin [16,21,22,23] were tested. However, the lack of prognostic sensitivity and specificity for individual markers, as well as marker-specific confounding parameters (like sepsis or organ failure), hamper the translation into clinical routine algorithms [2,24]. Compared to ’conventional’ protein-based markers, circulating miRNAs harbor several advantages: Circulating miRNAs are extraordinarily stable towards conditions that would usually degrade most proteins in serum or blood [25]. In addition, miRNAs do not have known postprocessing modifications, and with their size, as well as their chemical composition, they are much less complex than most other biological biomarkers [26]. Therefore, many authors have hypothesized that circulating miRNAs might perform better in the detection of sepsis or prognosis prediction in critically ill patients. As an example, miR-150 levels were found to be down-regulated in patients with urosepsis [27], as well as in a cohort of critically ill patients comprising various disease etiologies and severities [12]. Besides miR-150, miR-122, miR-133a, miR-146a, and miR-223 were identified to be indicative of the patients´ fate during and after ICU treatment [10]. However, their low sensitivity and specificity has deferred the use in clinical routine until now. In this context, we now provide the first multimarker, miRNA-based score for prognosis prediction in critical illness and sepsis. Importantly, our miRNA panel is biologically plausible as it incorporates biomarkers involved in inflammation and infection, key components of the pathophysiology of critical illness: We recently described alterations of miR-133a, miR-150, miR-155, and miR-193b* concentrations after cecal pole ligation and puncture-induced sepsis in mice [9]. Moreover, a dysregulation of miR-143 and miR-223 was found in leukocytes after LPS administration, as well as in various inflammatory diseases [28]. miR-122 and miR-192 represent liver-specific miRNAs with a specific dysregulation in hepatic injury and failure, representing a key feature of organ malperfusion associated with critical illness and sepsis [11]. In the present manuscript, we demonstrate that, besides miR-143, levels of all these miRNAs are altered in critical illness, supporting the selection of this 7-miRNA panel. Interestingly, within these miRNAs, only miR-133a, miR-143, and miR-223 were found to be regulated with respect to patients’ ICU survival, while only miR-133a and miR-150 were regulated with respect to patients’ overall survival, highlighting that individual miRNAs might specifically indicate distinct features of critical illness. It therefore seems likely that the integration of these miRNAs into a score might provide a more comprehensive picture, and therefore also provide a more accurate estimation of patients’ prognosis compared to the analysis of a single miRNA in this setting.

Despite important advances in the field of miRNA research, it is important to note that the inter-study variances in terms of miRNA regulation patterns are enormous, and different studies have shown even opposing results with respect to the deregulation of miRNA levels in critical illness and sepsis [15,29,30]. Though miRNAs are very stable molecules in serum or plasma, it is likely that a lack of standardization regarding sample collection, data normalization, and analysis might have biased certain studies. In an attempt to avoid theses biases, in the present study sample, collection and handling followed strict protocols, e.g., by using spiked-in RNA (SV-40) for normalization, which is regarded as the ‘gold standard’ by most authors [31,32,33]. Finally, the cut-off values for our score were defined using the broadly accepted Youden’s index method, leading to certain differences with respect to cut-off values from previous studies [9,11,12,15]. These measures were taken to overcome technical challenges of miRNA analysis from serum or plasma, giving rise to the expectation that circulating miRNAs might become novel, highly attractive biomarkers. Their value as prognostic biomarkers might be further increased through combination with other clinical markers, which is supported by the fact that integrating the patient’s age into our score further increased the predictive accuracy. Such scoring systems might help to stratify patients into those with a need for intensifying treatment measures and those with a more favorable prognosis that might, e.g., be treated in non-intensive care settings and are not in need of intensive treatment modalities usually applied in medical ICUs.

In summary, our study is the first to demonstrate the prognostic value of an miRNA-based scoring system in ICU patients. High scores upon admission were closely associated with increased ICU and long-term mortality, and lower scores reliably indicated a favorable prognosis. Our data therefore provide evidence for a potential role of serum miRNA concentrations, and especially for combining different serum miRNA levels as an innovative prognostic tool in critically ill patients. Such data might help to improve prognostic assessments in critically ill patients upon admission to the ICU and should provoke further research to validate our results in larger and prospective studies on critically ill patients.

## Figures and Tables

**Table 1:** Baseline characteristics of the study population.

Parameter	All Patients
Number	204
Sex (male/female)	132/72
Age median (range) [years]	63 (18–89)
APACHE-II score median (range)	16 (2–40)
SAPS2 score median (range)	43 (8–79)
ICU days median (range)	7 (1–83)
Death during ICU [%]	22.1
Ventilation [%]	64.7
Body mass index median (range) [kg/m^2^]	26.1 (16.6–86.5)
Creatinine (range) [mg/dL]	1.3 (0–15)
WBC median (range) [×10^3^/µL]	12.2 (0.1–67.4)
CRP median (range) [mg/dL]	100 (<5–230)
Procalcitonin median (range) [µg/L]	0.71 (0–180.6)
Interleukin-6 median (range) [pg/mL]	105 (0–83,000)
INR median (range)	1.18 (0–9.2)

APACHE, Acute Physiology and Chronic Health Evaluation; CRP, C-reactive protein; ICU, intensive care unit; INR, international normalized ratio; SAPS, simplified acute physiology score; WBC, white blood cell count.

**Table 2:** Disease etiology of the study population.

	Sepsis	Non-Sepsis
	*n* = 127	*n* = 77
**Sepsis critical illness** Source of infection n (%)		
Pulmonary	67 (52.8)	
Abdominal	28 (22.0)	
Urogenital	3 (2.4)	
Other	29 (22.8)	
**Non-sepsis critical illness** n (%)		
Cardiopulmonary disease		26 (33.8)
Decompensated liver cirrhosis		11 (14.3)
Non-sepsis other		40 (51.9)

**Table 3:** Comparison of the data from the current analysis with the respective published data.

	Mir-122		Mir-133a		Mir-143		Mir-150		Mir-155		Mir-192		Mir-223	
	Published [11]	204 Patients Cohort	Published [9]	204 Patients Cohort	Published * [19]	204 Patients Cohort	Published [12]	204 Patients Cohort	Published [13]	204 Patients Cohort	Published [14]	204 Patients Cohort	Published [15]	204 Patients Cohort
**Patients vs. Control**	↑	↑	↑	↑	=	=	=	=	↑	↑	↑	rauf	≈(↓)	↓
**Sepsis vs. Non-Sepsis**	=	=	↑	↑	=	=	=	=	=	=	=	=	=	=
**ICU Survival vs. Non-Survival**	=	=	↓	↓	↑	↑	=	=	=	=	=	=	↑	↑
**Overall Survival vs. Non-Survival**	=	=	↓	↓	=	=	↑	↑	=	=	=	=	=	=

* Disease markers, *in pres.*

## Supplementary Materials

The following are available online at https://www.mdpi.com/2077-0383/8/10/1644/s1.
