# Supplementary material for: A Combined Score of Circulating miRNAs Allows Outcome Prediction in Critically Ill Patients
Source: J Clin Med. 2019 Oct 9;8(10):1644. doi: 10.3390/jcm8101644 (PMC6832199; doi:10.3390/jcm8101644)
Supplement: Supplementary file 1 [file jcm-08-01644-s001.ppt]

## Slide 1
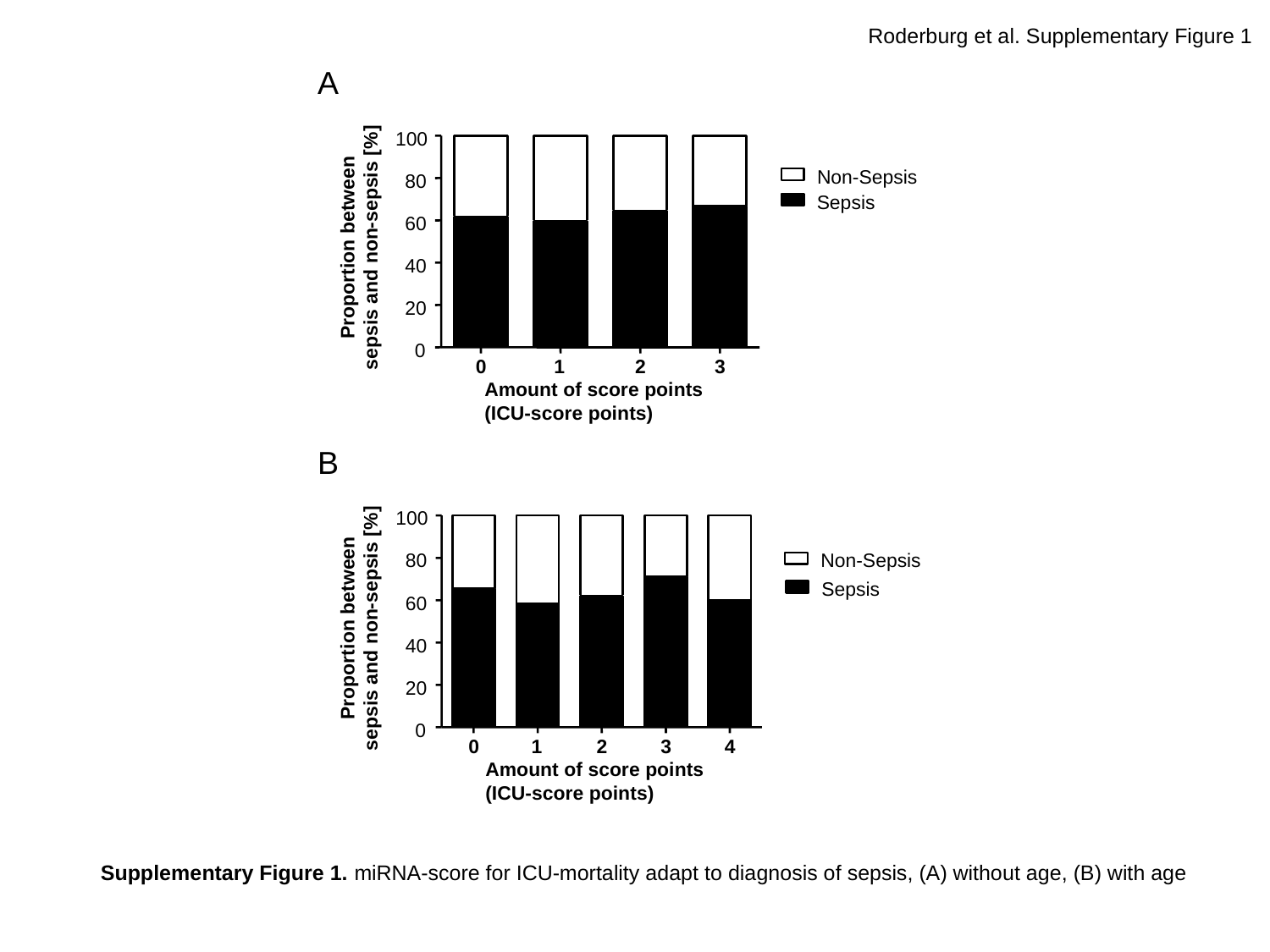

Roderburg et al. Supplementary Figure 1
A
100
Non-Sepsis
80
Sepsis
60
Proportion between
sepsis and non-sepsis [%]
40
20
0
0
1
2
3
Amount of score points
(ICU-score points)
B
100
80
Non-Sepsis
Sepsis
60
Proportion between
sepsis and non-sepsis [%]
40
20
0
0
1
2
3
4
Amount of score points
(ICU-score points)
Supplementary Figure 1. miRNA-score for ICU-mortality adapt to diagnosis of sepsis, (A) without age, (B) with age
